# Transactions of the Semi-Annual Meeting of the Med. Society of Erie County, N. Y.

**Published:** 1854-07

**Authors:** 


*Transactions of the Semi-Annual Meeting of the Medical Society of the County of Erie, N. Y.*

The semi-annual meeting of the Medical Society of the county of Erie, was held in this city, at American Hall, on Tuesday the 13th of June, 1854, commencing at 10½ o'clock, A. M. The President, Dr. J. G. House, of Springville, presiding.

Some forty-five gentlemen were present during the session and participated in the business of the meeting.

Applications for membership with the society, were received from Drs. Richard W. Nelson, C. F. Gay, Austin W. Nichols, Frederick Gardner, Joel Underhill, William A. Newell, Chauncey B. Hutchins, Charles B. Richards, and Edward Stork, all of whom, after a proper examination of their credentials, were admitted upon their compliance with the By-laws of the society.

Dr. Treat, from the Committee on Purchase of Books,

*Reported*, That the publications of the Sydenham Society had been ordered, but that they had been lost in their passage to this country, in consequence of the wreck of the vessel at sea, and that a second set had been ordered but had not yet been received.

The Committee on Society Dinner, through Dr. Ring,

*Reported*, That it had, upon conference with many members of the society, been deemed best to defer this festivity until the January meeting. The report was accepted, and on motion to that effect, the committee was continued, to carry out the objects of its appointment, at the next annual meeting.

The Committee on the Tariff of Prices for appointments and medical services for the county of Erie, through Dr. Samo, its chairman, presented an able report, treating the subject in all its bearings at considerable length.

After a note of acceptance, it was, on motion,

*Resolved,* That the list of prices therein contained be taken up *seriatim* for adoption.

Each article was thoroughly discussed, and the following prices, as a *minimum compensation*, for the services specified, established by the *unanimous vote* of the members present:

| | |
|---|---|
| For Physician to the Alms House, per year, in addition to which, the residence of an intelligent student or graduate, . . | $600 00 |
| " Physician to the Penitentiary, per year, . . . . . | 300 00 |
| " " " Jail, . . . . . . . . . | 150 00 |
| " attendance on the poor of the city, *for District No.* 1, (including all east of Main st. and south of Genesee st. to the city limits,) . . . . . . . . . . . | 250 00 |
| *District No.* 2, (all west of Main st. and bounded on the north and west by the old city limits,) . . . . . . . | 125 00 |
| *District No.* 3, (all east of Main st. and north of Genesee st. to the city limits,) . . . . . . . . . . | 125 00 |
| *District No.* 4, (all not included in the foregoing, taking in the town of Black Rock,) . . . . . . . . . | 100 00 |
| Attendance on Coroner's Inquests:— | |
| For post-mortem examinations, . . . . . . . | 10 00 |
| " vewing the body, . . . . . . . . . . | 5 00 |
| Mileage to be charged according to the fee bill of the Buffalo Medical Association. | |
| Health Physician of the city of Buffalo, per year, . . . | 1000 00 |

The above prices to be for services only, and exclusive of any medicines.

On motion of Prof. White, it was

*Resolved,* That the report of the committee, and the action of the society thereon be published in the Buffalo Medical Journal, and in the daily and weekly papers of the city.

Prof. White also offered the following resolution:

*Resolved,* That it shall be dishonorable for any member of this society, to accept any appointment, or perform any services contained within the foregoing Tariff of Prices, at a less sum than therein specified. *Adopted unanimously.*

On motion of Dr. Wilcox, it was

*Resolved*, That the secretary furnish a copy of the Tariff of Prices, with the resolutions relating thereto, to the board of supervisors, superintendents of the poor, the coroners and the common council of the city of Buffalo.

Toward the close of the morning session, Prof. Hamilton introduced to the notice of the society, a patient suffering from "an enormous adipose tumor of the neck," with the following sketch of the history of the case:

Louis Derome, æt. 50 years, born in Montreal, C. E., of French extraction, a boat builder, at which trade he has worked since he was fourteen years old, and until three years ago, when on account of the growth of the tumor, he was obliged to suspend labor altogether.

About fourteen years since when shaving one morning, he noticed a fullness under the chin, especially on each side, upon which he remarked that he was growing fat. From that time there has been a steady increase to the present day.

It now measures thirty-two inches around the neck, completely encircling the neck, and extending from his chin to four inches below the top of the sternum, and covering all the space between the occiput and the back, looking at a distance as if the neck was encircled with a large muffler. It is composed of masses varying somewhat in shape and solidity.

There is a roll of fat forming also just below this mass, across the shoulders. The breasts are gradually becoming penduluos with adipose deposit, as well as the belly. There are, moreover, two distinct and remarkable tumors on the back of the haunches, of the same character. In all other parts of his body the deposit of fat is spreading.

His health is uninterruptedly good. He suffers no pain, or sense of discomfort in the tumors, nor has he at any time, except that when he labors, the mass behind the neck becomes congested and tense, and throws the head forward into an uncomfortable position, thus completely disabling him from active exertion.

At the opening of the afternoon session, Prof. Hamilton read an article on *Fractures of the Humerus and their Treatment*, questioning the propriety of the present usual mode of dressing the same with the arm in a flexed position; and presented a patient just recovered from that injury.

On motion of Dr. Wallis, a vote of thanks was tendered Prof. Hamilton, for his able presentation of the subject to the society, and a copy requested for preservation among its papers.

The following resolutions were offered by Prof. White:

*Resolved,* That the thanks of this society be presented to Messrs. Putnam, of the State Senate, and Germain and Weed, of the Assembly, for their humane and successful efforts, during the last session of the Legislature, in behalf of the bill for the promotion of the study of Human Anatomy.

*Resolved,* That a copy of this resolution, signed by the officers of this society be presented to those gentlemen.

They were unanimously *adopted.*

The *Oration* was then delivered by Dr. S. B. Hunt, the orator of the day. *Subject—The cranial characteristics and powers of the Human Races.*

At its conclusion, on motion of Prof. Hamilton, a vote of thanks was tendered the orator for his able address, and a copy requested for publication in the Buffalo Medical Journal, and for preservation in the archives of the society.

Dr. John Boardman was appointed orator for the annual meeting in January next.

The society then adjourned. J. M. N.

---

*The New Tariff of Fees.*—We call the attention of our readers abroad, as well as those resident in the county of Erie, to the report upon the compensation for medical services in the public charities.

This is no mere matter of local interest. The profession throughout the state are, so far as our knowledge extends, in need of some such action; and we the more cheerfully give publicity to this excellent report, in the hope that other county societies may take similar steps toward placing themselves upon an equality with the general public in this regard.

The report itself embodies all the argument necessary upon the subject. We can add nothing to it, and we would much less take anything from it. The vote in the county society gave most decisive evidence that Dr. Samo, and his colleagues in the committee, only expressed a sentiment so general as to be unanimous.

The only objection raised to any part of the report, in the full and earnest debate which preceded its adoption, was a fear that the prices named were rather too low, than too high. The attendance at the meeting was more than usually large,.and it was evident from the spirit manifested, that this action was to be decisive so far as any member of the county society was concerned; and it is not at all probable that any member of the profession will incur the disgrace of violating the spirit or letter of the resolutions as finally adopted. H.

*Medical Society of the County of Erie.*
June 13, 1854.
*Abstract from Minutes.*

The Committee on *Tariff of Prices* for appointments and medical services for the county, through Dr. Samo,

*Reported,* Treating the subject in all its bearings at length, and in a most masterly manner.

" After the acceptance of the report, it was on motion of Dr. White,

"*Resolved,* That the list of prices therein contained be taken up *seriatim* for adoption.

"The report was then taken up re-read, and each article therein thoroughly discussed, and voted upon.*

" On motion of Dr. Hamilton, the report of the committee, together with the fee bill, (as amended) was *unanimously adopted.*

" On motion of Dr. White, it was

"*Resolved,* That the report of the committee, and the action of the society thereon be published in the Buffalo Medical Journal and in the daily and weekly papers of the city.

" Dr. White also offered the following resolution:

"*Resolved,* That it shall be dishonorable for any member of this society to accept any appointment, or perform any services contained within the foregoing tariff of prices, at a less sum than therein specified. *Adopted unanimously.*

" On motion of Dr. Wilcox, it was

"*Resolved,* That the secretary furnish a copy of the tariff of prices, with the resolutions relating thereto, to the board of supervisors, superintendents of the poor, the coroners, and the common council of the city of Buffalo."

" I hereby certify that the foregoing are correct copies of the proceedings had upon the subject therein embraced, and are faithful transcripts of the minutes of the proceedings of the Medical Society of Erie County, held at American Hall, Buffalo, June 13, 1854.

JAMES M. NEWMAN,
Secretary Medical Society, Erie Co."

---

*The report was adopted without alteration or amendment, except that the charge of *viewing* a body before coroner's jury was increased from $3 to $5, and the paragraph succeeding, " For each assistant deemed, &c.," was stricken out.

### *Report of the Committee on a Medical Tariff of Prices.*

At the annual meeting of the Erie County Medical Society for January, 1854, a resolution was unanimously adopted, appointing a committee of three to report a tariff of prices for medical services and appointments in the county of Erie and city of Buffalo.

These services and appointments are the following, to wit: Physician to the Almshouse, Physician to the Penitentiary, Physician to the Jail, Physician to the county poor in the city of Buffalo and in the country and county towns, Health Physician of the city of Buffalo, and medical services upon coroner's inquests.

In estimating the value of these, your committee have consulted with the different medical incumbents of these various offices, as well as with those who have performed their duties heretofore. They have also been guided, in a great degree, by the judgment of the profession generally, and by statistics obtained from various sources. Your committee, however, regret to say, there are a few points upon which they have been unable to obtain the requisite information, and that, in consequence, their report will, therein, be necessarily defective. They believe, however, with the exception alluded to, it will be found to embrace all that was contemplated in the resolution. It was intended to present, in tabular form, a statement, showing the average number of sick, per week, prescribed for during the past year, in the different institutions named, along with the compensation for the current year; and such other statements as might throw light upon the subject. But it was found difficult, if not impossible, in the time, to get such full and complete information on these heads as would make such a table useful. They shall, therefore, be obliged to give the few statistics they have collected in a less comprehensive form.

At the Almshouse, the compensation for the current year is $400 to the attending physician, and board, &c., to a student resident in the establishment. The physician visits twice a week, and oftener when requested. Besides the ordinary sick list, which is large, there is an insane department here, containing, at the present time, sixty lunatics. Your committee will be unable to present at this time, any further statistics of this institution. The fault is theirs, in having delayed their application for them till too late to procure them in time for this report.

At the Penitentiary the average number of sick per week prescribed for during the last six months, is twenty-four; the compensation for the current year $100.

At the Jail during the past five months, about five per week, will represent the average number prescribed for. Compensation for the current year, $75.

The city of Buffalo has been divided into four medical districts, and a physician for the poor therein, appointed to each. $400 is the compensation for the current year, which amount is divided pro rata among the physicians, according to the number of patients in the districts. The boundaries of these may thus be given: all east of Main street and south of Genesee street to the city limits, is one district, which for convenience of reference is here set down as No. 1. All west of Main street and bounded on the north and west by the old city limits, we shall call No. 2. All east of Main street and north of Genesee street to the city limits, No. 3. And all not included in the foregoing, taking in the town of Black Rock, forms the remaining district, which we shall denominate No. 4. For district No. 1, during six months past, the sum of the medical duties is thus given: 220 visits; sixty office prescriptions; six cases of obstetrics; one case of fractured clavicle; one amputation of great toe. For No. 2, in the same period of time, the medical duties have been, sixty-five visits; fifteen prescriptions; one case of obstetrics, and two toes amputated. For No. 3, during the same period, fifty visits and twenty-five office prescriptions. For No. 4, your committee are not informed of the precise amount of business, but have learned that it is less than in either of the other districts.

The services of a physician upon a coroner's inquest are *always* of more or less importance; the compensation for which, as hitherto allowed, has been undoubtedly inadequate. $5. for a post-mortem examinatian, with any amount of attendance upon courts of law as it may happen, and $2. for viewing a body, have been the moderate and modest charge of the physician. Moderate as these are, however, they have been disallowed in the majority of instances, and sums less in amount substituted by the committee to whom the subject is referred by the board of supervisors. Perhaps your committee may except cases where the account rendered is ignored and thrown out altogether; which we believe has always been the case, where a medical assistant at a post-mortem has had the temerity to send in *his* account for services. That coroners may have occasionally put the county to a few dollars unnecessary expense, is very possible; but the following resolution, in reference to this subject, adopted by a recent board of supervisors, seems to your committee both unsafe and unsound; even did the statute not make it *imperative* on the coroner, as we are informed it does, to call a physician in *every* case of holding inquest. The resolution referred to reads as follows: "Resolved, that in the opinion of this board, a due regard to the interests of the public does not require the coroners to call upon physicians in cases of holding inquests, as frequently as has been practiced by some of the coroners of this county. Resolved, further, that the several coroners be respectfully requested not to impose unnecessary expense upon the county, by calling physicians in cases of inquests, except where they have good reason to believe that the scientific knowledge of such physicians may be necessary for the furtherance of justice."

Your committee's humble opinion on this matter is, that there could be very few cases of inquest indeed, where the presence of a physician might be safely dispensed with: few where the ends of truth and justice and the interests of community may not be subserved by his opinion.

In the instance of medical services to the poor in the country and in county towns, your committee regret to say that their information upon the subject is insufficient; and that consequently they have not been able to determine on a rate of compensation. It is very desirable that some uniform standard should be fixed for these services, and adopted by the society.

The office of Health Physician for the city of Buffalo completes our list. The duties attached to this appointment are, especially during the prevalence of cholera, or of a contagious epidemic disease, like small pox, both onerous and responsible, and the compensation should be liberal. $900 is the amount allowed for the current year.

In constructing a tariff, like the following, your committee have endeavored to keep in view the interests of all concerned, viz: the physician and his patient; the profession; and the public at large. To the physician this is not merely a question of dollars and cents; it is a question that appeals to a laudable ambition, besides its moral aspect, viewed in relation to the conscientious discharge of his duty. To the poor patient it is of vital consequence. Where the inadequacy of his compensation, and, indeed, the illiberality of every feature of his appointment, make it a constant struggle between the conscience and the pocket of the physician, to discharge his duty to his patient faithfully, it is easy to see and to believe, how vitally the patient is concerned in a question like this. To the profession collectively, as individually, it is of interest, involving as it does, to some extent, its dignity and standing in community. Indeed, this subject was originated and is sustained by those whose position and circumstances, in and out of the profession, preclude the idea of their being applicants or competitors for these appointments. And finally, it cannot be unimportant, evident as it must be to every one that considers it, that whatever tends to elevate our professional standing, or to facilitate the acquisition of medical knowledge' must, to a certain extent, prove beneficial to the public at large, between which and our profession there can exist no real antagonism of interests.

The "Tariff of Prices" is as follows:

To Physician to the Almshouse, your committee think $600 a minimum compensation. In addition to which, the residence there, as at present, of an intelligent student or graduate, seems indispensable.

For the Penitentiary, $300 has been considered the minimum for the efficient performance of the duties of physician there.

For the Jail, $150 is submitted as a minimum.

For City Poor, District No. 1, $250; No. 2, $125; No. 3, $125; No. 4, $100; aggregate $600. All of the foregoing are regarded by your committee as *minimum* rates of compensation.

Coroner's Inquests—For post-mortem examination, $10; for viewing body, $5. Mileage to be charged according to the rate of the Buffalo medical fee bill.

The salary of the health physician of the city of Buffalo, though comparatively liberal, is scarcely commensurate with the importance and responsibility of the office. Your committee therefore place it at $1000 as the minimum.

It is to be understood, that these estimates have no reference, whatever, to the medicines, &c., which duty, it is hoped, the physician will be relieved from in future. Your committee have learned with satisfaction, that the plan of furnishing medicines at the expense of the county, has been adopted at the almshouse during the past and present year. At the jail and penitentiary, however, and among the city poor, it is still required of the physician. This should not be. To the physician, the jail, penitentiary and almshouse, are as hospitals, where he is to practice his art, and like them they should be furnished with ample medical and surgical armamentaria, in order that he may practice it advantageously to all concerned, viz., to himself and to his patient; to his profession and to the public at large. It is due alike to science and to humanity; and your committee cannot but regard it as a most mischievous economy, thus to add the duty of apothecary, to the arduous and responsible duties of physician. It were well sometimes to remember, how much is frequently depending upon the practical skill and knowledge of the physician. Is it not, therefore, desirable, by all means, that he should give his sole attention to his art, nor be fettered by any thing that may restrict his usefulness? It is a duty society owes to itself, that, in its public institutious, whether for the criminal or the pauper, the physician have all the appliances of his art furnished to his hands. It, that is society will be the gainer by it.

The aggregate amount of these estimates is $2,650, not including the amount paid for services in cases of inquest, nor for services to the poor in the country and county towns, which may swell the whole to something over $3,000, and which will thus express the cost to the county per year for medical services. It might afford a subject of curious, if not profitable speculation, to be able to contrast this with the amount of legal expenses paid every year by the county.

The necessity for a tariff of prices is recognized by the greater part, if not by all, of the profession of Erie county. It should be *unanimously* sustained. Its success may depend upon the unanimity with which it is upheld. To the younger members of ths profession, the question, " Will *all adhere* to it ? " is the first to suggest itself. It cannot be doubted, however, if this be understood as the expressed will of the Society, that any evasion or infraction of its provisions must be regarded as equally if not more dishonorable than an invasion or infraction of the " Fee-bill," and subject the offender to all the odium of such a proceeding.

The duties pertaining to these appointments, it is conceded, can be most efficiently discharged by the younger members of the profession. It is, therefore, more emphatically, for their benefit this measure is calculated; and the hope is expressed that upon them they will be devolved in future. The older members, and those who, by their circumstances and position seem especially qualified to uphold this measure, should give it their countenance and support; such as is due to every regulation that has for its object the honor and usefulness of the profession.

Your committee have now discharged their duty, though imperfectly. It remains with the Society to perform theirs. The time must soon arrive, under the rapid increase of our population, when it will become necessary to construct the tariff anew. To a future committee, then, these rough materials, hastily thrown together, may, at least, serve as a basis upon which to erect a more perfect structure.

All of which is respectfully submitted.

J. B. SAMO,<br>

C. C. WYCKOFF, } COMMITTEE.<br>

J. G. HOUSE,

